# Free-ranging pigs identified as a multi-reservoir of *Trypanosoma brucei* and *Trypanosoma congolense* in the Vavoua area, a historical sleeping sickness focus of Côte d’Ivoire

**DOI:** 10.1371/journal.pntd.0010036

**Published:** 2021-12-22

**Authors:** Barkissa Mélika Traoré, Mathurin Koffi, Martial Kassi N’Djetchi, Dramane Kaba, Jacques Kaboré, Hamidou Ilboudo, Bernadin Ahouty Ahouty, Minayégninrin Koné, Bamoro Coulibaly, Thomas Konan, Adeline Segard, Lingué Kouakou, Thierry De Meeûs, Sophie Ravel, Philippe Solano, Jean-Mathieu Bart, Vincent Jamonneau

**Affiliations:** 1 Laboratoire de Biodiversité et Gestion des Ecosystèmes Tropicaux, Unité de Recherche en Génétique et Epidémiologie Moléculaire, UFR Environnement, Université Jean Lorougnon Guédé, Daloa, Côte d’Ivoire; 2 Unité de Recherche « Trypanosomoses », Institut Pierre Richet, Bouaké, Côte d’Ivoire; 3 Unité de Recherche sur les Maladies à Vecteurs et Biodiversité, Centre International de Recherche-Développement sur l’Elevage en zone Subhumide, Bobo-Dioulasso, Burkina Faso; 4 Programme National d’Elimination de la Trypanosomiase Humaine Africaine, Abidjan, Côte d’Ivoire; 5 Intertryp, IRD, Cirad, Université de Montpellier, Montpellier, France; University of Cincinnati, UNITED STATES

## Abstract

**Background:**

The existence of an animal reservoir of *Trypanosoma brucei gambiense* (*T*. *b*. *gambiense*), the agent of human African trypanosomiasis (HAT), may compromise the interruption of transmission targeted by World Health Organization. The aim of this study was to investigate the presence of trypanosomes in pigs and people in the Vavoua HAT historical focus where cases were still diagnosed in the early 2010’s.

**Methods:**

For the human survey, we used the CATT, mini-anion exchange centrifugation technique and immune trypanolysis tests. For the animal survey, the buffy coat technique was also used as well as the PCR using *Trypanosoma* species specific, including the *T*. *b*. *gambiense* TgsGP detection using single round and nested PCRs, performed from animal blood samples and from strains isolated from subjects positive for parasitological investigations.

**Results:**

No HAT cases were detected among 345 people tested. A total of 167 pigs were investigated. Free-ranging pigs appeared significantly more infected than pigs in pen. Over 70% of free-ranging pigs were positive for CATT and parasitological investigations and 27–43% were positive to trypanolysis depending on the antigen used. *T*. *brucei* was the most prevalent species (57%) followed by *T*. *congolense* (24%). Blood sample extracted DNA of *T*. *brucei* positive subjects were negative to single round TgsGP PCR. However, 1/22 and 6/22 isolated strains were positive with single round and nested TgsGP PCRs, respectively.

**Discussion:**

Free-ranging pigs were identified as a multi-reservoir of *T*. *brucei* and/or *T*. *congolense* with mixed infections of different strains. This trypanosome diversity hinders the easy and direct detection of *T*. *b*. *gambiense*. We highlight the lack of tools to prove or exclude with certainty the presence of *T*. *b*. *gambiense*. This study once more highlights the need of technical improvements to explore the role of animals in the epidemiology of HAT.

## Author summary

Significant efforts to control human African trypanosomiasis (HAT) since the 1990’s have drastically reduced the prevalence of the disease. Its elimination as a public health problem is being achieved. World Health Organization now targets the interruption of transmission for 2030. However, potential animal reservoirs of *Trypanosoma brucei gambiense* (*T*. *b*. *gambiense*), the main agent of HAT, may compromise this ambitious objective. It is the case in the Vavoua historical focus in Côte d’Ivoire where HAT cases were still diagnosed in the early 2010’s. During a study conducted in this area, we scrutinized the trypanosomes circulating in pigs and people sharing the same environment using serological, immunological, parasitological and molecular tools. No HAT cases were detected. We showed that *T*. *brucei* s.l. and *T*. *congolense* actively circulated in free-ranging pigs. Even if no tools were sensitive and specific enough to unambiguously identify *T*. *b*. *gambiense* directly from biological samples, six isolated strains from pigs positive for trypanosomes were amplified for TgsGP, the only currently accepted *T*. *b*. *gambiense* specific molecular marker. The apparent discrepancies between the presence of *T*. *b*. *gambiense* in pigs despite the absence of human cases is discussed. These results stress the need for an efficient “molecular toolbox” to easily detect and identify *T*. *b*. *gambiense* in any animal it may infect.

## Introduction

African trypanosomiases are vector-borne parasitic diseases that affect both humans and animals. They continue to impact public health and socio-economic development mainly in remote rural areas in Sub-Saharan Africa. Human African trypanosomiasis (HAT), or sleeping sickness, exists in two forms depending on the subspecies of *Trypanosoma brucei* [1]. The acute form, due to *T*. *b*. *rhodesiense*, occurs in Eastern and Southern Africa, while the chronic form, due to *T*. *b*. *gambiense*, is rampant in Western and Central Africa, causing 98% of HAT cases [2]. While *T*. *b*. *rhodesiense* HAT is known to be zoonotic, the role of a domestic or wild animal reservoir in the *T*. *b*. *gambiense* HAT epidemiology is still under debate [3,4]. Given the 2018 historic threshold of 1,000 cases reached [2], the potential role of animals as reservoirs did not prevent to control HAT so far. Nevertheless, it might compromise the interruption of transmission targeted by World Health Organization (WHO) for 2030.

Other species and subspecies of trypanosomes such as *T*. *b*. *brucei*, *T*. *congolense* and *T*. *vivax* are pathogenic to animals and cause Animal African Trypanosomiasis (AAT) or nagana. Economic losses due to AAT are measured in billions of dollars [5,6]. Human and animal trypanosomes share the same cyclical vectors in Sub-Saharan Africa, i.e. the tsetse flies (genus *Glossina*). An integrated management approach, based on the One health concept [7], is thus recommended for a sustainable elimination of African trypanosomiases [8,9].

In Côte d’Ivoire, HAT elimination as a public health problem is being achieved [2,10]. However, studies conducted in the two HAT foci that are still endemic in the central-western part of the country (Bonon and Sinfra) recently demonstrated that pigs were particularly infected by trypanosomes, although infections with *T*. *b*. *gambiense* could not be proven due to a lack of reliability of the tools used [11]. In the same region, the Vavoua HAT focus was epidemic at the end of the 1970’s [12–14] and thanks to both medical and vector control efforts [15,16], less than five HAT cases were reported yearly at the end of the 1990s [17]. Only four cases were still passively diagnosed in the early 2000s. However, the last two cases that were reported in 2011 and 2012 [18] raised the question of the infection origin. The Vavoua region is known to be a route of cattle transhumance and a place for pig husbandry [19]. While *T*. *brucei* s.l. and *T*. *congolense* have been previously described in pigs in the beginning of the 1980’s [20], the current situation of AAT is completely unknown in that area.

The aim of the present study was to simultaneously investigate the presence of trypanosomes, and identifying those, in pigs and people sharing the same environment for a better understanding of the current epidemiology of African trypanosomiases in the Vavoua area and to implement adapted strategies for a sustainable elimination of HAT.

## Material and methods

### Ethics statement

The human active screening was conducted within the framework of medical surveys and epidemiological surveillance activities supervised by the HAT National Control Program (HAT NCP). No sample other than those for routine screening and diagnostic procedures was collected. All participants were informed of the objective of the study in their own language and signed an informed consent form. Approval was obtained from the Ivorian national ethics committee, no.0308/MSLS/CNER-P.

No ethical statement is required by local authorities for domestic animal sampling. Any veterinarian may carry out blood sampling on domestic animals, with the authorization of the owner, as it is performed during prophylaxis or diagnostic campaign. Breeders gave their consent for animal sampling after being informed of the objectives of the study. For pig care, venous sampling was performed by a veterinary of the Laboratoire National d’Appui au Développement Rural (Ministry of Agriculture). A deworming treatment (Bolumisol, Laprovet) was provided for free to all pigs sampled.

### Study area

**Fig 1:**
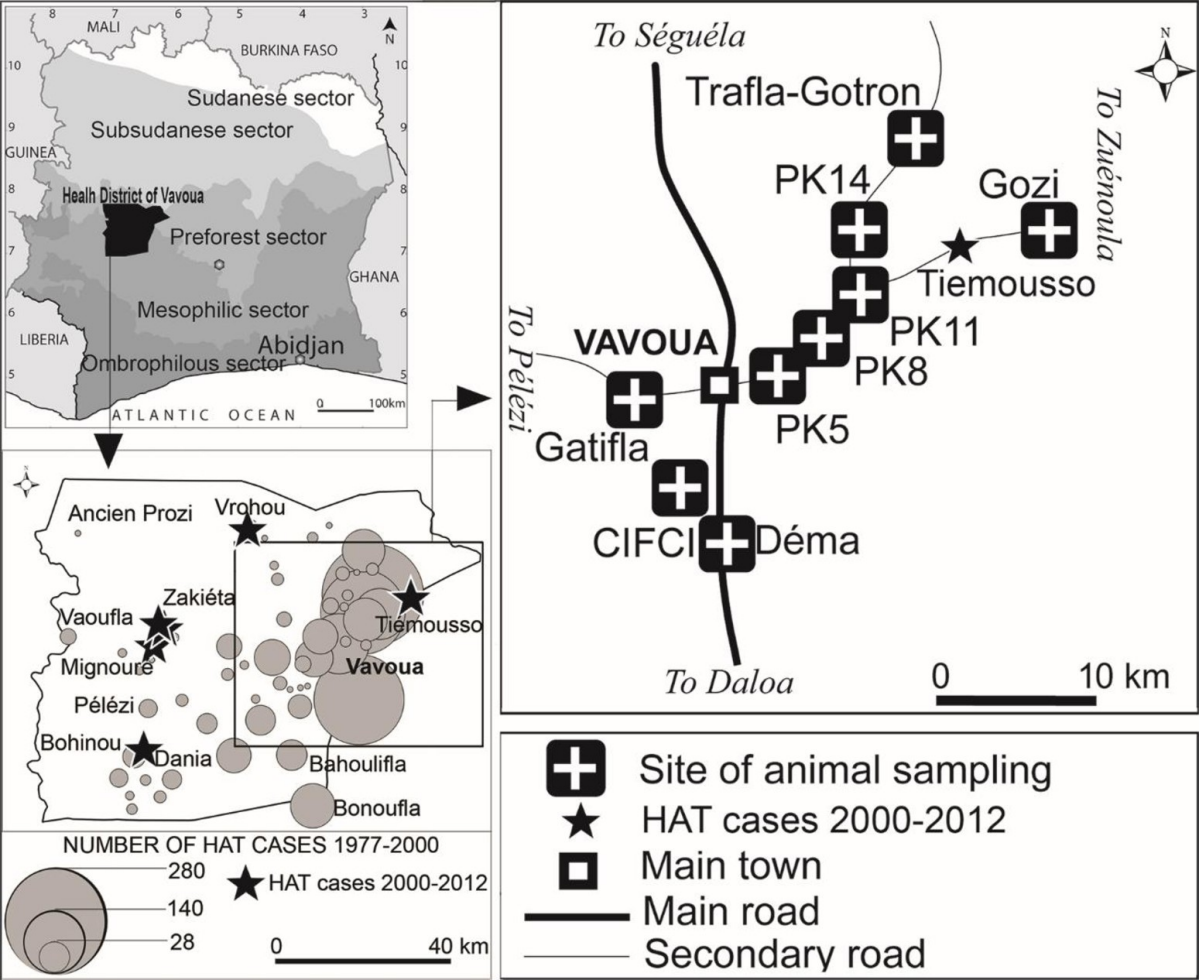
Study area and sites of animal sampling. The black stars represent the individual localization of the HAT cases diagnosed between 2000 and 2012. This figure was created by the mapping service of our team based at Institut Pierre Richet (Bouaké, Côte d’Ivoire).

The Vavoua area is located in the Haut-Sassandra region in the central-western part of Côte d’Ivoire (Fig 1). The vegetation was initially characterized by mesophilic forest with savannah inclusions mainly in the north. In the 1960’s, the forest has been progressively replaced by cash crops (mainly cocoa and coffee) leading to a favorable environmental context for HAT development in the area [14]. An epidemic situation broke out in the 1970’s. Fig 1 describes the number and the distribution of the HAT cases reported between 1977 and 1999 and the villages of residence of the six last cases reported since 2000. Nine study sites were selected in the most affected area where the presence of pigs was confirmed during a preliminary investigation.

### Field survey and sampling

The field survey was conducted in February 2017. For the human medical survey, we targeted pig’s breeders, their families and people living in the close neighborhood (considered as population at risk). Individuals who gave their informed consent were tested using the Card Agglutination Test for Trypanosomiasis (CATT, provided by Institute of Tropical Medicine Antwerp, Belgium) serological test [21] performed on blood (CATT-B) collected by finger prick. For CATT-B positive persons, 5mL of blood were collected in heparinized tubes and a twofold plasma dilution series in CATT buffer was tested to assess the highest dilution that was still positive on plasma (CATT-P). All positive CATT-P at a dilution of 1:4 or higher underwent parasitological investigations by direct examination of a lymph node aspirate and/or mini-anion exchange centrifugation technique (mAECT, provided by Projet de Recherches Cliniques sur la Trypanosomiase, Daloa, Côte d’Ivoire) [22] performed with 350 μL of buffy coat (BC) as previously described [23]. From these subjects, 1 mL plasma and 500 μL BC were aliquoted from the remaining blood and immediately stored at -20°C for further testing with PCR and immune trypanolysis (TL).

For the animal survey, considering *T*. *brucei* s.l. prevalence previously observed in the neighboring areas [11], we had scheduled to randomly test 20 pigs per study site. Prior to field survey, objectives of the study were explained to the inhabitants and local authorities of each village. After obtaining their approval, the farmers captured pigs, aged of more than one year whenever possible. For each captured pig, 9 mL blood were taken from the jugular vein in heparinized tubes. These blood samples were tested with the standard buffy coat technique (BCT) [24]. In the absence of accurate field serological diagnostic tests for African trypanosomiases in domestic animals and taking into account the limited sensitivity of BCT, we also used the CATT and mAECT following the same procedures as HAT diagnosis, to increase the possibility of both *T*. *b*. *gambiense* and animal trypanosomes detection. CATT-B was performed for each pig and CATT-P was performed for CATT-B positive pigs as described above. Only CATT-P at a dilution of 1:4 or higher were considered as CATT-P positive. The mAECT was performed with 350 μL of BC only for CATT-B positive pigs. In addition, 1 mL plasma and 500 μL BC were aliquoted from the remaining blood of all pigs and immediately stored at -20°C for subsequent PCR and TL testing. Information regarding the husbandry method (in pen or free-ranging pigs) and study sites were collected.

### Mice infection and isolation of trypanosomes

Trypanosome isolation in the field was performed by intraperitoneal inoculation of 0.5 mL whole blood from each pig positive to BCT and/or mAECT, in two Naval Medical Research Institute (NMRI) mice (produced in CIRDES, Bobo-Dioulasso, Burkina-Faso, from paternal strains purchased from Charles River laboratories, France). These were immunosuppressed with cyclophosphamide (300 mg/kg of Endoxan) administered before inoculation and then every 5 days. Five days post inoculation, parasitemia was determined daily by direct microscopic examination (X400) of mouse tail blood [25]. When parasitaemia reached 32.10^6^ parasites/mL, blood was collected by cardiac puncture of mice as previously described [26] and parasites were separated from mice blood using mAECT. Purified parasites were centrifuged (1200 g for 10 min) and parasite pellets were stored at -20°C for subsequent PCR analysis.

### Molecular diagnosis

DNA from 500 μL of BC was extracted using the DNeasy Blood and Tissue kit (Qiagen, Valencia, CA, USA) following manufacturer’s instructions. DNA from the isolated strains was extracted according to the same process, after having re-suspended the parasite pellets in 200 μL PBS. Negative extraction controls were systematically included during the process. PCR amplification was performed using specific primers for *T*. *brucei* s.l. (TBR1-2) [27], *T*. *congolense* savannah type (TCS1-2) [28], *T*. *congolense* forest type (TCF1-2) [28] and *T*. *vivax* (TVW1-2) [29]. The TgsGP1/2 primers [30] targeting the TgsGP gene specific of *T*. *b*. *gambiense* [31] were used in a single round PCR on all BC positives to TBR1-2 PCR and all isolated strains. All isolated strains were also tested for TgsGP presence with nested primers (TgsGPsense2 and TsgGPantisense2) [32]. The PCR reactions were carried out in a thermocycler (Eppendorf Mastercycler nexus) in 25 μl final volume, containing 1 X Qiagen HotStarTaq Master Mix, 20 pmol of each primer and 5 μl of DNA sample. The PCR products were visualized by electrophoresis in a 2% agarose gel (MP Biomedical, Eschwege, Germany) stained with Gel Red (Interchim, Montluçon, France) and illuminated with UV light.

### Trypanolysis test

Both human and animal plasma samples were processed with the immune trypanolysis test (TL) using cloned populations of *T*. *b*. *gambiense* variant antigen type (VATs) LiTat 1.3, LiTat 1.5 and LiTat 1.6 as previously described [33]. LiTat 1.3 and LiTat 1.5 VATs are supposed to be specific for *T*. *b*. *gambiense*, while LiTat 1.6 VAT is expressed in *T*. *b*. *gambiense* and *T*. *b*. *brucei* [34].

### Data management and statistical analysis

We compared the detection power for the different techniques and in the different situations (sites, husbandry method) with Fisher’s exact tests when possible under the R commander package (rcmdr) [35,36] for R [37], or with 100,000 randomizations in R when necessary, except for CATT-P for which we used a Krukal-Wallis test with rcmdr. This resulted in many *p*-values presented in supporting information. We corrected for the false discovery rate (FDR) with the Benjamini and Hochberg (BH) procedure [38], with the p.adjust command in R. Husbandry method only varied in the site PK14 and was only tested there. To analyze the concordance between serological and molecular data obtained from free-ranging pigs, a Venn diagram was built using a freely available online software (http://bioinformatics.psb.ugent.be/webtools/Venn).

## Results

### Human medical survey

In total, 345 subjects were tested ranging from 0 in Trafla Gottron (TG) (subjects refused to be sampled) to 98 in PK5 (Table 1). Serological examination revealed 15 CATT-B positive individuals and 6 CATT-P≥¼ (2 in PK5, 3 in Gatifla and 1 in Dema) for whom parasitological investigations, TBR PCR and TL were negative.

**Table 1:** Serological and parasitological results of human survey.

Study sites	Persons tested	CATT-B+	CATT-P≥1/4	PI, TBR PCR or TL+**
CIFCI	7	0	0	0
Dema	25	1	1	0
Gatifla	62	3	3	0
Gozy	3	0	0	0
PK5	98	5	2	0
PK8	91	5	0	0
PK11	34	1	0	0
PK14	25	0	0	0
TG*	0	0	0	0
Total	345	15	6	0

*TG = Trafla Gottron

**PI, TBR PCR or TL+ = Parasitological investigations, PCR using the TBR primers or Trypanolysis test positive

CATT-P was performed when CATT-B was positive. Similarly, confirmation tests (PI, TBR PCR or TL) were performed for CATT-P≥1/4 suspects as described in the Material and Methods section.

### Animal survey

A total of 167 pigs were tested and the complete database is given in S1 Table. This sampling consisted on 70 pigs in pen in PK5, PK8, PK11 and PK14; and 97 free-ranging ones in CIFCI, Dema, Gatifla, Gozy PK14, and TG (Table 2). Mix husbandry was only practiced in PK14 and we included an equivalent number of pigs in pen and free-ranging pigs.

**Table 2:** Distribution of tested pigs according to study sites and the husbandry method.

Study sites	Pigs in pen	Free-ranging pigs	Total
CIFCI	0	15	15
Dema	0	17	17
Gatifla	0	25	25
Gozy	0	22	22
PK5	20	0	20
PK8	20	0	20
PK11	20	0	20
PK14	10	10	20
TG*	0	8	8
Total	70	97	167

*TG = Trafla Gottron

**Fig 2:**
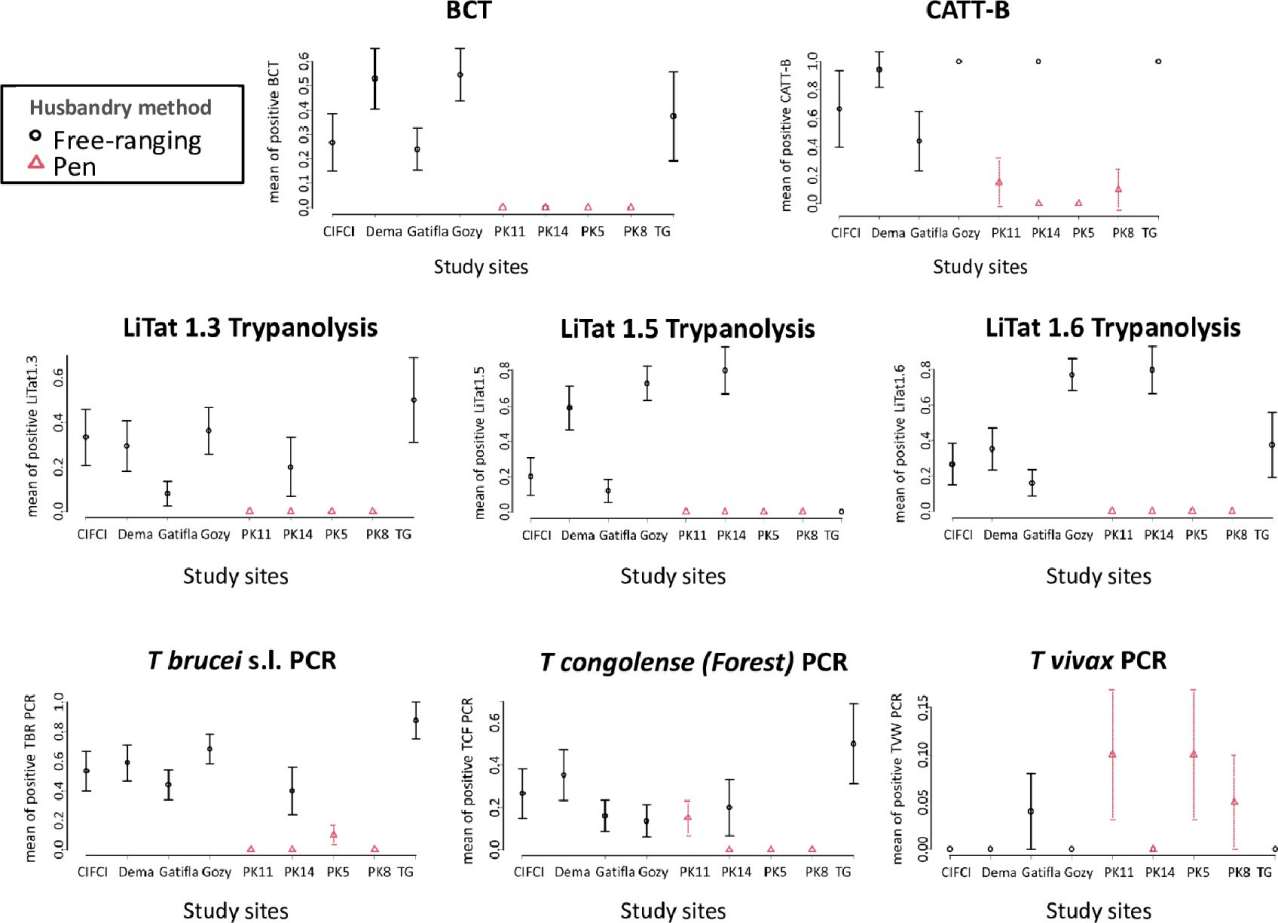
Diagnostic tests results regarding the study sites and the husbandry methods. Complete data are available in supporting information. All means are given with 95% confidence intervals. BCT = Buffy Coat Technique, CATT-B = Card Agglutination Test for Trypanosomiasis performed on blood, TG = Trafla Gottron.

TCS and TgsGP single round PCR were negative for all BC tested. Global positivity rates with the other tests ranged from 4% (TVW PCR) to 49% (CATT-B). We compared the BCT, CATT-B, TL and PCR results regarding the study sites and the husbandry methods (Fig 2). In PK14, the only site where both husbandry methods were applied together, free ranging pigs were more often positive for CATT-B (p-value<0.0001), LiTat1.5 (p-value = 0.0014) and LiTat1.6 (p-value = 0.0014) (S2 Table).

Except for TVW PCR (p-values>0.5), study sites, and probably the husbandry method, together had a highly significant impact on the diagnostic tests results (all p-values<0.003) (S3 Table). As can be seen in Fig 2, TG and Gozy (and to a lesser extent Dema, Gatifa and CIFCI), sites with exclusive free ranging pigs, represented the most heavily infected sites. The sites with the lowest prevalence were those of pen husbandry. We thus decided to focus on the free ranging sites for the rest of the analysis.

Serological and parasitological results obtained in the field for the 97 free-ranging pigs according to the study sites are detailed in Table 3. The average prevalence was 35% using the BCT, ranging from 24% (Gatifla) to 55% (Gozy) except for PK14 where all BCT were negative. The global seroprevalence using the CATT-B was 79% ranging from 44% in Gatifla to 100% in Gozy, PK14 and TG. Almost all the CATT-B positive pigs (91%) were CATT-P≥1/4. Unfortunately, mAECT could not be performed for 18 CATT-B positive pigs in CIFCI (8), Dema (8) and Gatifla (2) study sites due to test supply issues. The global prevalence obtained with the mAECT performed on 57 CATT-B positive pigs was 93%, ranging from 50% in CIFCI to 100% in Gatifla and TG. mAECT was significantly more sensitive than BCT (*p* ≤ 0,001) that was only positive for 17 out of the same 57 pigs (30%). The difference was remarkable in PK14 where 9 out of the 10 pigs BCT-negative were mAECT-positive. Taking into account both BCT and mAECT results, the global prevalence in free-ranging pigs was 72% (70/97). A significant difference was observed between the study sites for all tests. CIFCI and Gatifla appeared to be less affected by trypanosome infections than the other sites (Table 3).

**Table 3:** Serological and parasitological results of free-ranging pigs according to study sites.

Study sites	Nb pigs	BCT+	CATT-B+	CATT- P≥1/4	mAECT
CIFCI	15	4 (27%)	10 (67%)	9 (90%)	1/2** (50%)
Dema	17	9 (53%)	16 (94%)	15 (94%)	5/8** (63%)
Gatifla	25	6 (24%)	11 (44%)	10 (91%)	9/9** (100%)
Gozy	22	12 (55%)	22 (100%)	19 (86%)	21 (95%)
PK14	10	0 (0%)	10 (100%)	10 (100%)	9 (90%)
TG*	8	3 (38%)	8 (100%)	7 (88%)	8 (100%)
Total	97	34 (35%)	77 (79%)	70 (91%)	53/57 (93%)
P-value		0.014	<0.001	<0.001	0.036

*TG = Trafla Gottron

**The number of mAECT performed are given since the test cannot be performed on all CATT B positive pigs

Pig numbers (Nb pigs), and number of positive animals for BC, CATT-B, CATT-P and mAECT, as described in the Material and Methods section, are given.

**Fig 3:**
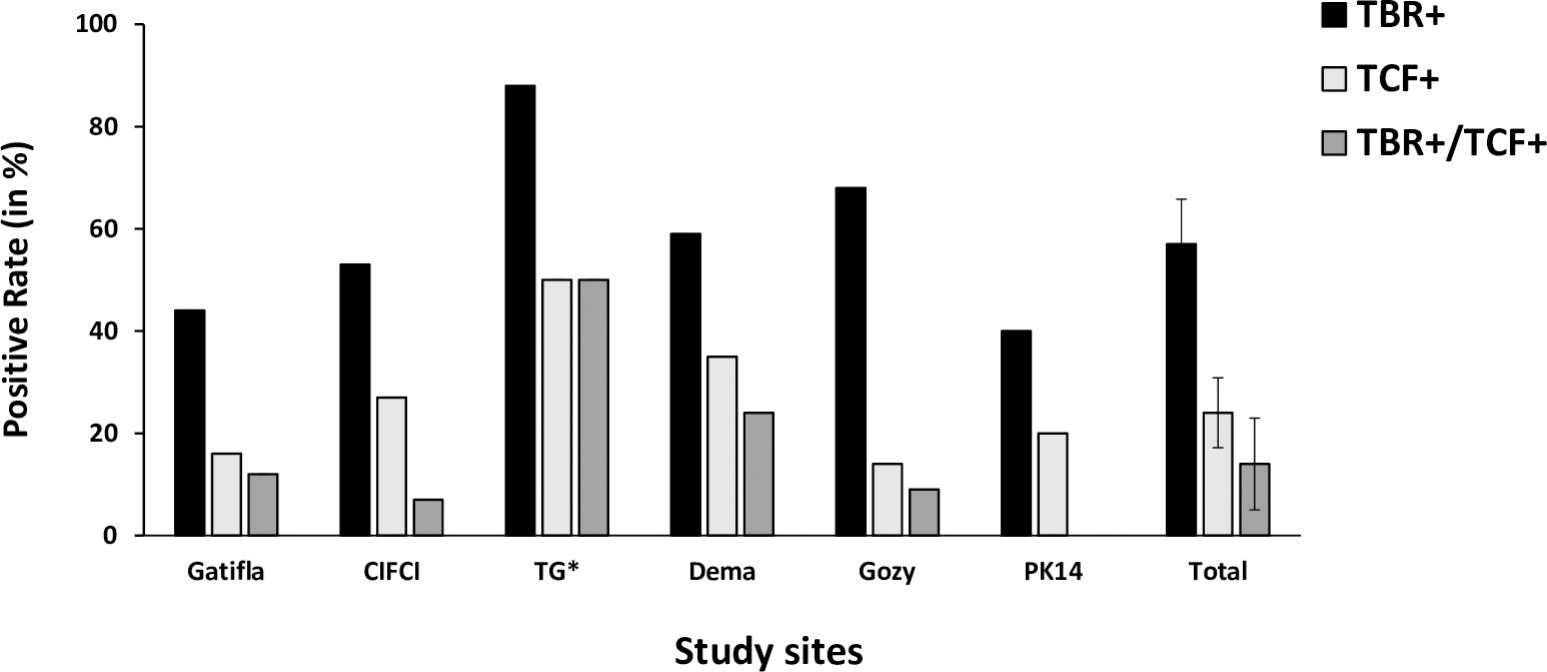
PCR results of free-ranging pigs according to study sites. TBR = TBR1-2 primers, TCF = TCF1-2 primers, + = positive test * TG = Trafla Gottron.

**Fig 4:**
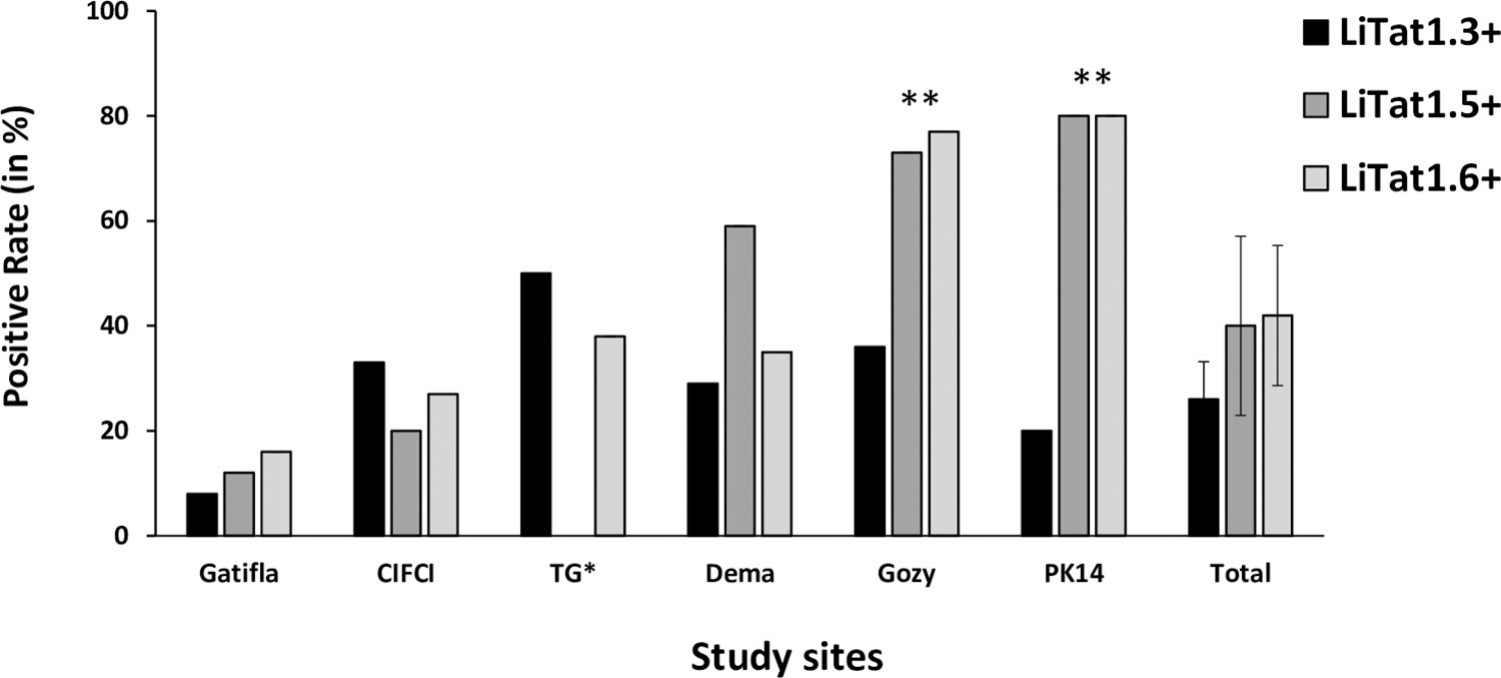
TL results of free-ranging pigs according to study sites. + = positive test * TG = Trafla Gottron ** = LiTat 1.5 and 1.6 occurrence was significantly higher (p values<0.001) in PK14 and Gozy comparing to the other study sites.

**Fig 5:**
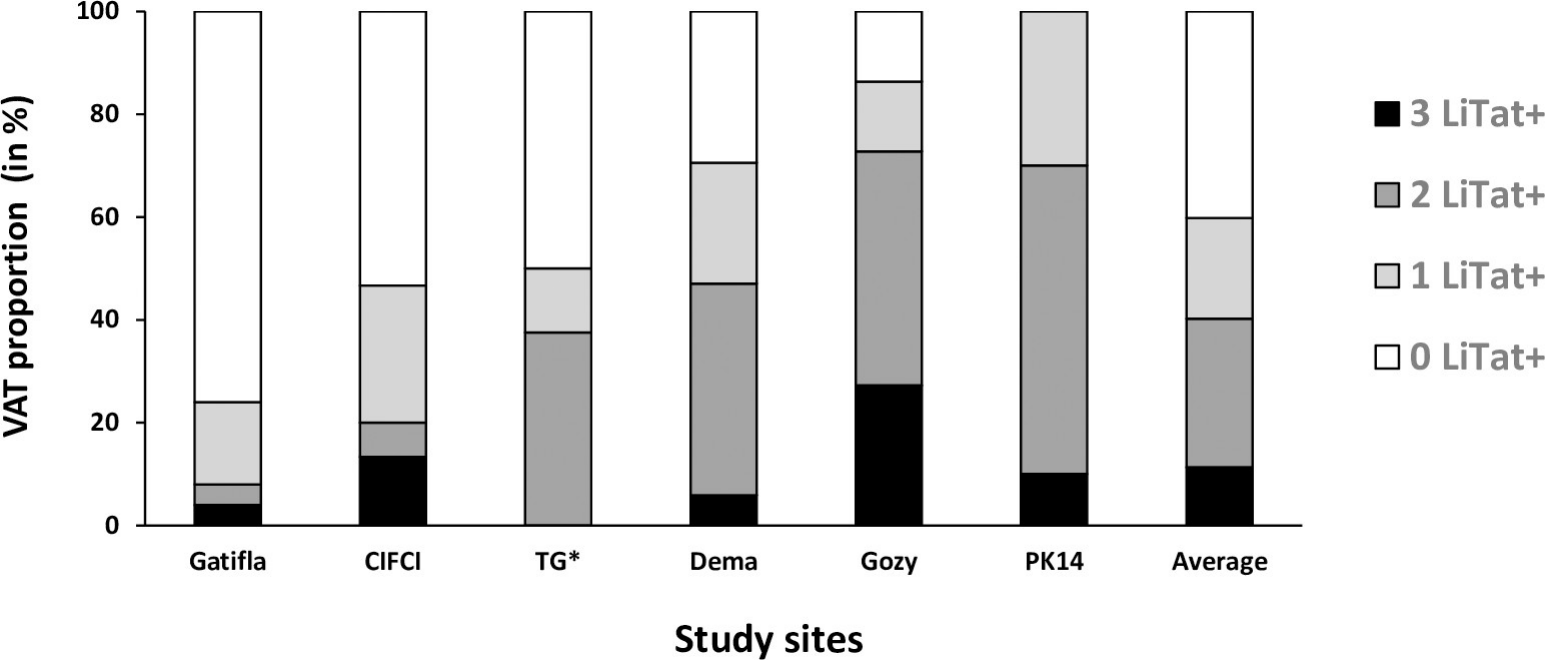
LiTat combined-profiles in the free-ranging pigs according to the study sites. + = positive test * = TG = Trafla Gottron.

The PCR results obtained for the 97 free-ranging pigs are given in Fig 3. They show higher global positivity rate for TBR PCR (57%) than for TCF ones (24%) with 14% of the pigs positive with the two PCR. Results were very heterogeneous between the study sites but no significant differences were observed (Fig 3). For TL results (Fig 4), the global positivity rate for LiTat 1.6 was the highest (43%) ranging from 16 to 80% according to the study site, followed by LiTat 1.5 (41%) ranging from 0 to 80% and LiTat 1.3 (27%) ranging from 8 to 50%. Significant differences were observed between the study sites for LiTat 1.5 and LiTat 1.6 mainly due the high positivity rates observed in Gozy and PK14 (between 70 and 80%), the low positivity rate of LiTat 1.5 and LiTat 1.6 in Gatifla and the absence of LiTat 1.5 in TG. No significant differences were observed between the study sites for LiTat 1.3. Fig 5 shows the global proportions of free-ranging pigs positive to 3 (11%), 2 (29%) and 1 VAT (20%) and the proportions according to the study sites, once more confirming an important heterogeneity between those. All the combinations of TL positive results with the three VAT were observed, but the combination Litat 1.3 negative, LiTat 1.5 positive and LiTat 1.6 positive was the most represented profile with 18 pigs (19%).

### Isolation of trypanosome strains and PCR results

**Fig 6:**
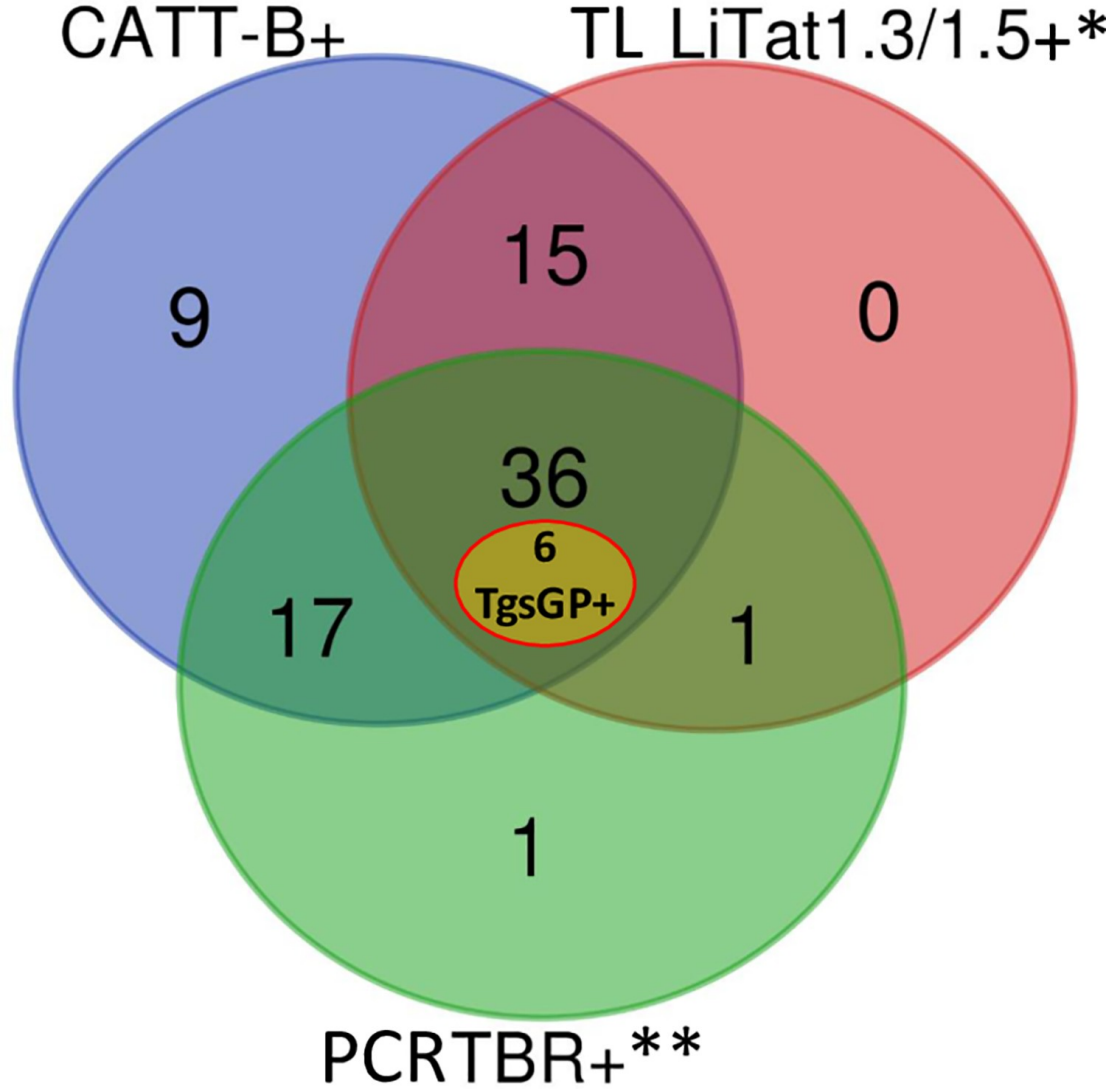
Venn diagram comparing congruence between serological and molecular data from free-ranging pigs. CATT-B = Card Agglutination Test for Trypanosomiasis performed on blood, * For TL (trypanolysis) data, LiTat 1.3 and 1.5 (considered as *T*. *b*. *gambiense* specific) results were fused into one dataset to simplify the visualization and interpretation of the diagram. TgsGP PCR data were added manually. ** PCR TBR (TBR1-2 primers) performed on blood samples.

A total of 22 strains were isolated, stabilized and purified (Table 4). They were all negative to TCS and TVW PCR. However, 19 (86%) and 9 (41%) strains were positive to TBR and TCF, respectively. Positive results with both PCR were observed for 8 strains (36%) suggesting that mixed infections are common. Two strains could not be amplified for any of the primers. Only one strain from Gozy was clearly positive after a single round TgsGP PCR (S1 Fig). The nested TgsGP PCR gave strongly positive bands for five additional strains from Gozy (2), PK 14 (2) and TG (1) (S2 Fig). The TgsGP PCR products of these six positive samples were sequenced and resulted in 270 nucleic acids showing 100% homology with the sequence of TgsGP gene (Gene Bank accession number FN555988) (S3 Fig). Surprisingly, two of these strains were TBR PCR negative, while the six corresponding blood samples were TBR PCR positive (Fig 6). Moreover, five other strains that gave a low intensity band with the single round TgsGP PCR (S1 Fig) were negative for the nested TgsGP PCR. The corresponding PCR products could not be sequenced due to a limited amount of product.

**Table 4:** PCR results on isolated strains from free-ranging pigs.

Study sites	No Isolated strains			PCR targets		
		TBR +	TgsGP +	NTgsGP +	TCF +	TBR + /TCF+
Dema	2	2	(1)	0	0	0
Gatifla	3	3	(1)	0	1	1
Gozy	8	6	1 (1)	3	3	2
PK14	4	3	0	2	2	2
TG*	5	5	(2)	1	3	3
Total	22	19 (86%)	1 (5%)	6 (27%)	9 (41%)	8 (36%)

*TG = Trafla Gottron

The numbers in brackets correspond to weakly positive results

NTgsGP = Nested TgsGP

Number of strains positive for TBR, TgsGP, NTgsGP and TCF targets, and those positive for both TBR and TCF targets, are given.

### Concordance between serological and molecular analyses

Finally, we proposed to evaluate the congruence of the serological and molecular results regarding the diagnosis of *T*. *brucei* s.l. in blood and plasma. We focused on CATT-B, TL and TBR PCR data obtained from the 97 free-ranging pigs (Fig 6). A strong overall congruence was observed with 87.3% (69/79) of the samples positive for at least two methods and 45.6% for the three (36/79). Only one sample out of 55 TBR PCR positive samples (1.8%) did not exhibit a positive serology and 72% (40/55) of the TBR PCR positive samples were positive for both CATT-B and TL tests. It is interesting to stress that for the six TgsGP-positive strains, the three tests performed on blood and plasma gave positive results. Among the 70 pigs in pen, 63 were negative for the three methods, five and two were only CATT-B and TBR PCR positive, respectively.

## Discussion

In Côte d’Ivoire, One Health approaches are being conducted to adapt strategies for reaching HAT sustainable elimination. A previous study conducted in the Bonon and Sinfra HAT endemic foci concluded that pigs were the most trypanosome-infected domestic animals, but the presence of *T*. *b*. *gambiense* could not be proven [11]. The present study was carried out in the Vavoua historical focus where the last two HAT cases were diagnosed in the early 2010s and where pig husbandry is widespread. One objective was to evaluate the possible human contamination from a pig transmission cycle in a historical HAT focus. Based on our results, this hypothesis seems unlikely since no HAT cases nor TL-positive subjects were observed in the sampled people living in close vicinity of the pigs. However, with the decline in incidence of HAT, this disease is no longer considered as a threat in the study area. Thus, we cannot exclude the existence of undetected human cases.

The global results obtained with BCT, TL and PCR confirmed high trypanosome infection rates in pigs as already observed in previous studies conducted on the neighboring Bonon and Sinfra HAT endemic foci [11,39] or in other study areas in West and Central Africa as in Nigeria [40], Chad [41] or Cameroon [42–44]. Such high infection rates in pigs may be linked to their tolerance to trypanosomes as already observed in the field [20] or during experimental infections with *T*. *b*. *gambiense* [45], and certainly illustrate that the pig is a preferential host for tsetse flies [46,47].

An originality of this work was to compare the results obtained regarding the husbandry methods of pigs. We show that free-ranging pigs were significantly more trypanosome-infected than pigs raised in pen including in the PK14 site where the two husbandry methods are practiced. This is probably due to the fact that free-ranging pigs, roaming freely in the humid and shady areas and in the protected sacred forests near by villages, are highly exposed to *Glossina palpalis* that shares the same biotope as already observed [11,46]. Pigs have already been described as a preferential feeding host for this tsetse species [46] that have recolonized the studied area after the end of the vector control campaigns conducted in the 1980’s [15,19]. These proximity and trophic interactions result on predominant free-ranging pig/tsetse transmission cycles in the vicinity of villages. The low infection rates observed in pigs in pen could be because pigsties are located inside or close to the villages far away from the tsetse flies favorable biotopes, and are thus probably less exposed.

We decided to focus our diagnosis analysis on the six free-ranging pigs study sites with an overall prevalence of 35% observed with BCT and of 72% when combining BCT with mAECT. Our results confirmed those recently observed in Bonon and Sinfra [11] with the highest PCR positivity rate obtained for *T*. *b*. *brucei* (more than half of the pigs are positive) followed by *T*. *congolense* forest type (a quarter positive) and with more than 10% of mixed infections with these two species. The unexpected presence of *T*. *vivax* in pigs raised in pen advocates for alternative life cycles, with the putative involvement of tabanids insuring mechanical transmission. With the increase of molecular approaches, presence of *T*. *vivax* in pigs has been recently reported [48,49] contrasting with the historical data previously reviewed [50].

In this study, we also used the CATT and mAECT initially adapted for HAT diagnosis, to increase the possibility of *T*. *b*. *gambiense* detection. The CATT-B test showed high positivity rates up to 100% in three free-ranging pig sites. Most of the CATT-B positive pigs were CATT-P ≥ 1/4 confirming the important reactivity with the test. More than 90% of the mAECT performed on CATT-B positive pigs were positive. These results confirm that the CATT is not specific of *T*. *b*. *gambiense* due to cross-reactions with other trypanosomes as already observed [51–54]. The SD Bioline HAT rapid diagnostic tests (RDT) [55], another serological test initially developed for HAT diagnosis, also showed a lack of *T*. *b*. *gambiense* specificity when used in animals [41,56,57]. In absence of serological tests adapted for field diagnosis of African trypanosomiases in animal surveys, we propose the use of CATT as screening methods, despite their lack of *T*. *b*. *gambiense*-specificity.

The mAECT, known as the most sensitive parasitological test for *T*. *b*. *gambiense* detection in human [58,59], was applied on CATT positive pigs for which infection rates from 50% to 100% were observed. mAECT was much more sensitive than BCT. Although the buffer pH and ionic strength conditions have been adapted for *T*. *b*. *gambiense* detection in humans [60], mAECT seems nevertheless suitable to detect other trypanosomes such as *T*. *b*. *brucei* or *T*. *congolense* forest type in pigs with similar sensitivity as PCR but without the possibility to identify the trypanosome species limiting its additional value regarding the study of the animal reservoir of *T*. *b*. *gambiense*.

Because the tools mentioned above could not be used to specifically identify the presence of *T*. *b*. *gambiense* in the studied pigs, the rest of our investigations focused on the TgsGP PCR and TL results, because they are reported to be *T*. *b*. *gambiense* specific. No pig was positive with the TgsGP PCR performed on BC. The lack of sensitivity of this PCR targeting a single copy gene [30,31] was recently confirmed in an experimental study [61]. We then cannot exclude false negative results (negative PCR despite the presence of *T*. *b*. *gambiense*) especially when using a single round PCR as done in this study. This was a deliberate choice regarding the single round and nested PCR results firstly obtained on purified DNA from isolated strains. With the single round PCR, a band of low intensity was observed for several strains at the expected size but the PCR product could not be sequenced to confirm it corresponded to the TgsGP sequence. The nested TgsGP PCR applied on these samples were negative suggesting a non-specific amplification during the first PCR round. On the other hand, negative samples for the single round PCR were unambiguously positive for the nested PCR. Only one strain was clearly positive for both the single round and the nested PCR. For this sample, the presence of the TgsGP was confirmed by sequencing. If we take into account all the TgsGP PCR positive results (single round or nested), *T*. *b*. *gambiense* was detected in 50% of the isolated strains (11% free-ranging pigs). This was inconsistent with both the *T*. *b*. *gambiense* prevalence in human in the area and the results of the human survey performed in the frame of this study.

We may suspect that the nested TgsGP PCR could gave false positive results, for two main reasons. First, we cannot exclude a contamination during amplification workflow even if all experiments were validated by negative controls. Such contamination may have occurred in any of the laboratories that routinely work with *T*. *b*. *gambiense* DNA, mainly when using nested PCR to increase sensitivity. Second, a TgsGP-related gene (Tb10.v4.0178) that shared 81% homology with TgsGP has been described in *T*. *b*. *brucei* [62,63]. Even with *T*. *b*. *gambiense* specific primers, a risk of *T*. *b*. *brucei* non-specific amplification should be considered when PCR is forced. In a recent study targeting TgsGP to detect *T*. *b*. *gambiense* in tsetse flies in a very low HAT prevalence area in Uganda, the TgsGP primers were suspected to cross-react with DNA from an “unidentified source” after sequencing the DNA products [64]. Non-specific TgsGP PCR products could explain the reported presence of *T*. *b*. *gambiense* in domestic and wild animals in foci where no or very few HAT cases were reported [41,65,66]. This might also explain the TgsGP positive results recently observed on animals in the Mandoul focus in Chad [57], an area where tsetse densities were significantly reduced by a vector control campaign [67]. Alternatively we cannot exclude that TgsGP nested PCR may detect very low level of DNA in animals, that does not mean any active transmission to humans.

The *T*. *b*. *gambiense* specificity is also questioned regarding the LiTat 1.3 and LiTat 1.5 TL that gave respectively up to 50% and 80% positive results according to the study site. More than 10% of the free-ranging pigs were positive with the two VAT. The LiTat 1.3 gene was already observed in *T*. *b*. *brucei* isolated from pigs in Côte d’Ivoire [68] and positive TL results were observed in cattle in *rhodesiense* HAT and AAT endemic region in Uganda [56]. Further experimental studies are required to confirm or not the *T*. *b*. *gambiense* specificity of this method.

Despite the doubts observed in this study regarding the TL and TgsGP PCR *T*. *b*. *gambiense* specificity, one isolated strain for which a strong PCR signal was observed with both the single round and nested TgsGP PCR is more likely to be *T*. *b*. *gambiense*. The corresponding free-ranging pig was positive to TBR PCR (on both BC and isolated strain) and to LiTat 1.5 and 1.6 TL (not 1.3). We then cannot exclude the existence of free-ranging pig/tsetse/*T*. *b*. *gambiense* transmission cycles in which humans are usually not involved, except if an “accidental” infection occurs when an infective tsetse bites a human. This may explain the infection of the last HAT cases reported in the neighboring villages where a human/tsetse transmission cycle is very unlikely regarding the low prevalence of the disease. A long-term residual human reservoir could also explain these infections as already suspected, especially in the neighboring foci of Sinfra and Bonon [33].

Studies on the *T*. *b*. *gambiense* animal reservoir are also complex because of the diversity of the epidemiological contexts of HAT foci. Animal reservoirs can involve wild animals with a very important diversity of species [65,69–71]. Regarding domestic animals, it depends on the population customs, the animal species raised and the husbandry methods. If free-ranging pigs seems to be mostly involved in trypanosome circulation in Côte d’Ivoire, other animal species appeared to be involved in other countries with differences observed between different HAT foci [44,57] and even within the same focus [42]. In our study, we observed significant differences in prevalence and in result profiles for several diagnostic tests according to the study sites that are only a few kilometers apart. Environmental factors probably influenced the pig/tsetse contact and the transmission of the different trypanosome species. Ecological and entomological aspects have not been included in our study design and could limit the interpretation of our data. Another limitation concerns the lack of data collection on both the human and pig population sizes, number of pig breeders and number of pigs per breeder in each study sites. Further studies on a larger and more exhaustive sample would allow a more accurate evaluation of the role of pigs in African trypanosomiases epidemiology.

Another difficulty relies on the fact that the most exposed animal(s) are often multi-infected as already observed in several studies [11,72]. This was clearly illustrated in the present one with the high prevalence of *T*. *brucei* s.l. and *T*. *congolense* forest type mixed infections. The association between these two species, already mentioned in other studies [11,20,39,51,72] may hold epidemiological consequences that would deserve further investigations. The important heterogeneity observed regarding the TL profile results according to the study sites suggested the likely circulation of different *T*. *brucei* s.l. strains containing different VAT and mixed infections with several strains in single pigs. The congruence observed between the serological (CATT-B and TL) and molecular (TBR PCR) results showed that despite their limited specificity regarding the trypanosome species, these tools appeared to be useful for further field investigations on animal trypanosomiases.

In summary, although all the diagnostic tests performed in this study on plasma, blood and isolated strains showed high trypanosome prevalence in free-ranging pigs, none was able to prove with certainty the presence or absence of *T*. *b*. *gambiense*. TL and TgsGP PCR may even tend to overestimate *T*. *b*. *gambiense* prevalence, which would represent an undesirable bias. This study once more highlights the need for technical improvements in exploring the epidemiological role of animals in HAT. Studies focusing on the deeper genetic knowledge of the circulating strains (by NGS methods) and phenotypical evidences related to resistance toward human serum are currently conducted to solve these questions. Excluding or evidencing this role using field and experimental studies is crucial to adapt the control strategies and to define indicators to reach the interruption of transmission targeted by WHO in 2030 [2]. Ignoring or under-estimating the importance of this topic may lead to repeat the same mistakes as for the Guinea worm for which the reporting of a canine reservoir has challenged and delayed the eradication process [73]. Free-ranging pigs obviously represent a potential HAT risk for humans, but may also constitute a potential source of contamination for other domestic animals as zebu cattle that are sensitive to trypanosome infections [74].

## Supporting information

S1 Table Complete database of the diagnostic results for the 167 pigs. ND = not down; + = positive;— = negative; (+) = positive TgsGP PCR with low intensity band.
(XLS)

S2 Table Diagnostic tests results for the 20 pigs in PK14 regarding the husbandry method. 0 = negative; 1 = positive.
(XLSX)

S3 Table Diagnostic tests results regarding the study sites. (XLSX)

S1 Fig Agarose gel obtained with single round TgsGP on isolated strains. M = molecular weight marker (100 bp DNA ladder) Sample 2 (pig 102) = positive with a clearly positive band Samples 1 (pig 82) and 3 (pig 86) = positive with a low intensity band C1+ and C2+ = positive PCR controls C- = negative PCR control.
(PPTX)

S2 Fig Agarose gel obtained with nested TgsGP on isolated strains. M = molecular weight marker (100 bp DNA ladder) Sample 3 (pig 66), 7 (pig 69), 8 (pig 84), 12 (pig 100), 14 (pig 102) and 16 (pig 104) = positive with a clearly positive band C1+ and C2+ = positive PCR controls (*T*. *b*. *gambiense* reference stock) C- = negative PCR control.
(PPTX)

S3 Fig Alignment of the sequences of the nested TgsGP positive samples with DAL972 reference stock. Sample 3 (pig 66), 7 (pig 69), 8 (pig 84), 12 (pig 100), 14 (pig 102) and 16 (pig 104) C1+ and C2+ = positive PCR controls (*T*. *b*. *gambiense* reference stock).
(PPTX)
