# Supplementary figures and images for: Free-ranging pigs identified as a multi-reservoir of Trypanosoma brucei and Trypanosoma congolense in the Vavoua area, a historical sleeping sickness focus of Côte d’Ivoire
Source: PLoS Negl Trop Dis. 2021 Dec 22;15(12):e0010036. doi: 10.1371/journal.pntd.0010036 (PMC8735613; doi:10.1371/journal.pntd.0010036)

## Slide 1
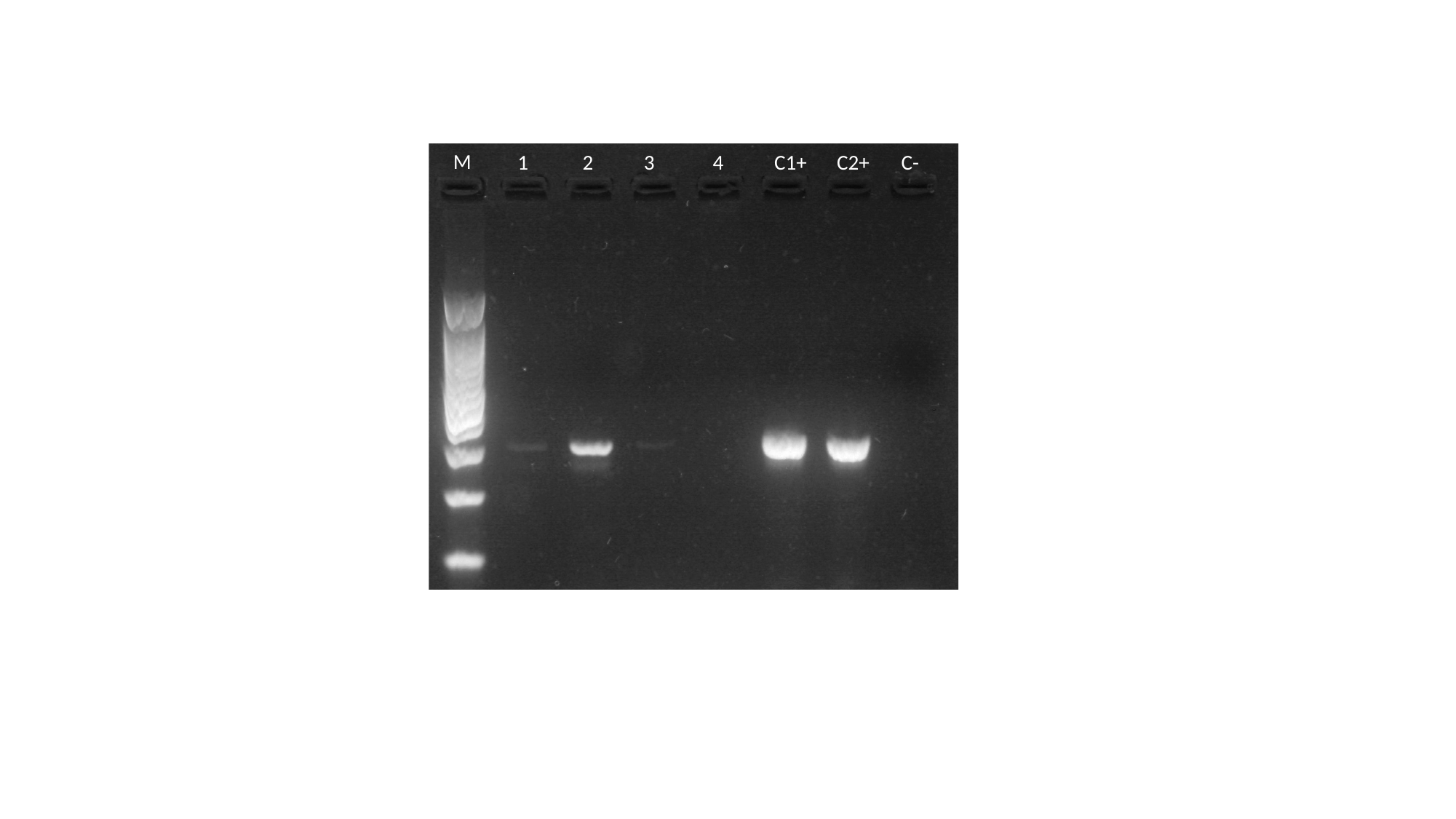

M
1
2
3
4
C1+
C2+
C-

Supplement: S1 Fig — M = molecular weight marker (100 bp DNA ladder) Sample 2 (pig 102) = positive with a clearly positive band Samples 1 (pig 82) and 3 (pig 86) = positive with a low intensity band C1+ and C2+ = positive PCR controls C- = negative PCR control. (PPTX) [file pntd.0010036.s004.pptx]

## Slide 1
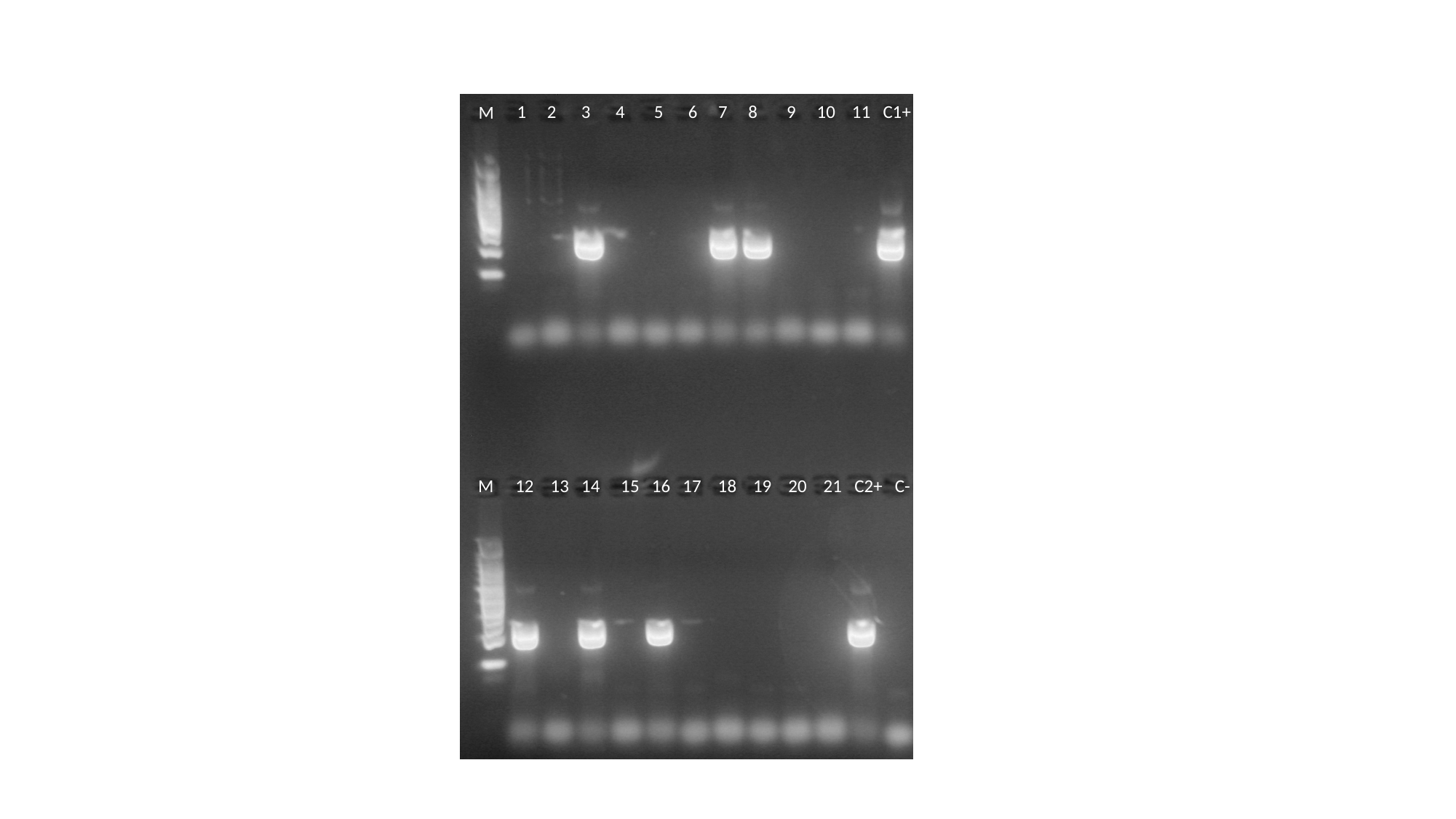

1 2 3 4 5 6 7 8 9 10 11 C1+
M
M
12 13 14 15 16 17 18 19 20 21 C2+ C-

Supplement: S2 Fig — M = molecular weight marker (100 bp DNA ladder) Sample 3 (pig 66), 7 (pig 69), 8 (pig 84), 12 (pig 100), 14 (pig 102) and 16 (pig 104) = positive with a clearly positive band C1+ and C2+ = positive PCR controls (T. b. gambiense reference stock) C- = negative PCR control. (PPTX) [file pntd.0010036.s005.pptx]

## Slide 1
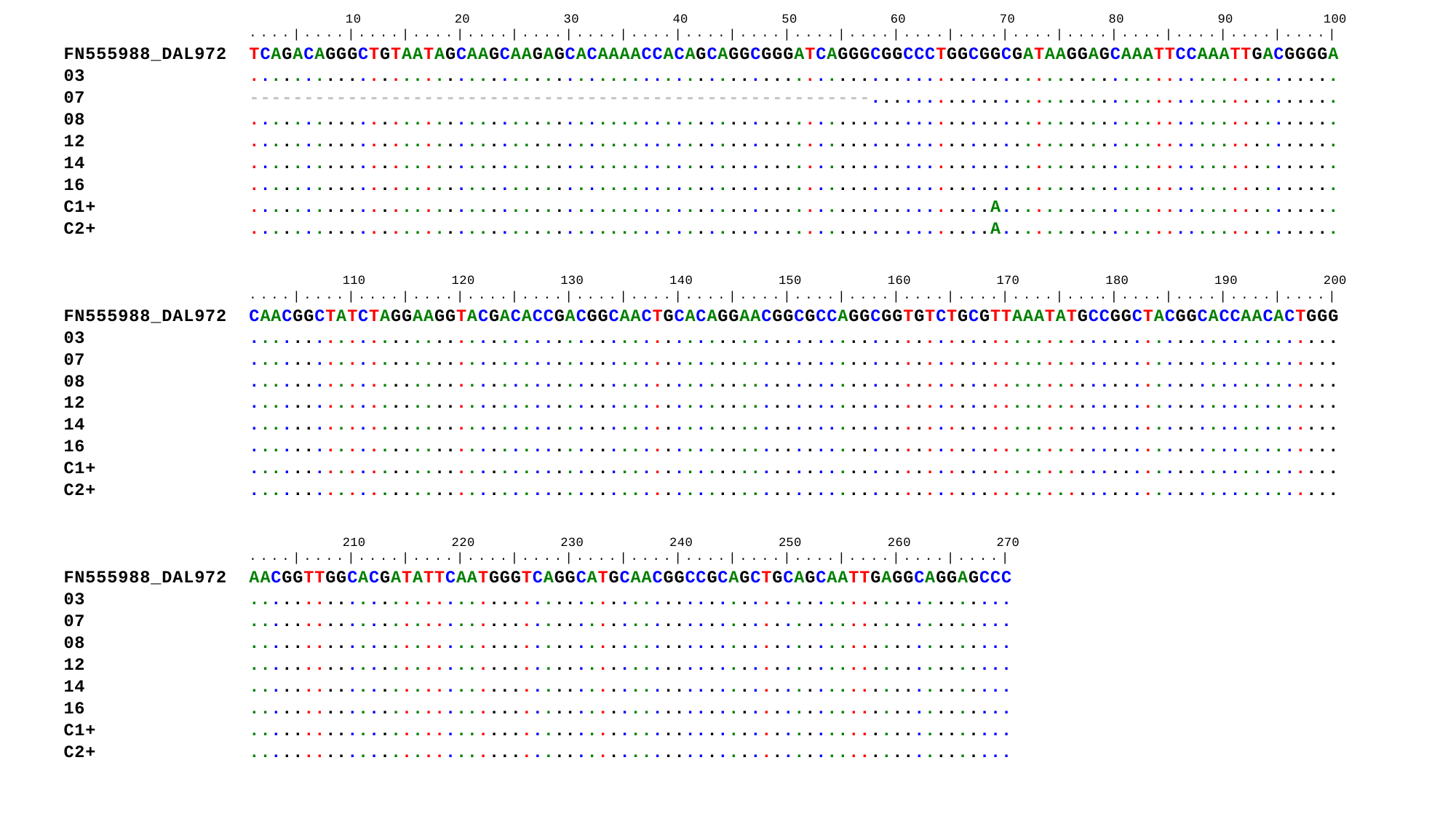

Supplement: S3 Fig — Sample 3 (pig 66), 7 (pig 69), 8 (pig 84), 12 (pig 100), 14 (pig 102) and 16 (pig 104) C1+ and C2+ = positive PCR controls (T. b. gambiense reference stock). (PPTX) [file pntd.0010036.s006.pptx]
